# Interplay between Heart Disease and Metabolic Steatosis: A Contemporary Perspective

**DOI:** 10.3390/jcm10081569

**Published:** 2021-04-08

**Authors:** Mohammad Said Ramadan, Vincenzo Russo, Gerardo Nigro, Emanuele Durante-Mangoni, Rosa Zampino

**Affiliations:** 1 Department of Precision Medicine, University of Campania “Luigi Vanvitelli”, 80138 Naples, Italy; mohammad.ramadan@unicampania.it; 2 Department of Translational Medical Sciences, AORN Ospedali dei Colli-Monaldi Hospital, 80131 Naples, Italy; vincenzo.russo@unicampania.it (V.R.); gerardo.nigro@unicampania.it (G.N.); 3 Cardiology Unit, AORN Ospedali dei Colli-Monaldi Hospital, 80131 Naples, Italy; 4 Infectious and Transplant Medicine Unit, AORN Ospedali dei Colli-Monaldi Hospital, 80131 Naples, Italy; rosa.zampino@unicampania.it; 5 Department of Advanced Medical and Surgical Sciences, University of Campania “Luigi Vanvitelli”, 80138 Naples, Italy

**Keywords:** non-alcoholic fatty liver disease, fatty liver, cardiovascular diseases, ischemic heart disease, atrial fibrillation, pathophysiology, metabolic syndrome, non-alcoholic fatty liver disease diagnosis, cardiovascular diseases prognosis, cardiovascular medications

## Abstract

The liver-heart axis is a growing field of interest owing to rising evidence of complex bidirectional interplay between the two organs. Recent data suggest non-alcoholic fatty liver disease (NAFLD) has a significant, independent association with a wide spectrum of structural and functional cardiac diseases, and seems to worsen cardiovascular disease (CVD) prognosis. Conversely, the effect of cardiac disease on NAFLD is not well studied and data are mostly limited to cardiogenic liver disease. We believe it is important to further investigate the heart-liver relationship because of the tremendous global health and economic burden the two diseases pose, and the impact of such investigations on clinical decision making and management guidelines for both diseases. In this review, we summarize the current knowledge on NAFLD diagnosis, its systemic manifestations, and associations with CVD. More specifically, we review the pathophysiological mechanisms that govern the interplay between NAFLD and CVD and evaluate the relationship between different CVD treatments and NAFLD progression.

## 1. Introduction

Non-Alcoholic Fatty Liver Disease (NAFLD) is currently thought to cause most cases of chronic liver disease (CLD) worldwide, accounting for as high as 75% of them in some studies [1]. NAFLD is currently recognized as the hepatic clinico-pathological manifestation of metabolic syndrome (MetS)—which also includes abdominal obesity, arterial hypertension, atherogenic dyslipidemia, and reduced insulin sensitivity, all of which were subsequently shown to be closely associated with NAFLD [2,3,4]. The prevalence of NAFLD is on the rise, with variable estimations ranging from 20% to 50% among adults in Western countries [5,6,7]. This rise should not be surprising, due to NAFLD association with MetS, and the current growing epidemics of MetS in the West [8]. Further strengthening the association with MetS, it was shown that NAFLD prevalence rises to 70–75% among individuals with type 2 diabetes (T2DM) and up to 95–99% in those with obesity [9,10]. Although NAFLD was initially identified as a fatty liver disease arising in the absence of significant alcohol intake, it has subsequently been increasingly correlated to a glucose and lipid metabolic derangement. Accordingly, an expert panel recently proposed to change nomenclature from NAFLD to ‘metabolic associated fatty liver disease’ (MAFLD) [11]. The relationship of NAFLD with visceral obesity and alterations in glucose (insulin resistance, diabetes mellitus) and lipid (hypertriglyceridemia, low HDL cholesterol, hypercholesterolemia) [12] metabolism implicates this disease in a large range of clinical conditions relevant not only to liver disease, but also cardiovascular disease (CVD), diabetes, and cancer, making NAFLD/MALFD an emerging topic in clinical medicine.

## 2. NAFLD Diagnosis: Current Approaches

NAFLD is a broad term comprising a wide spectrum of liver diseases, from simple steatosis or liver fat accumulation—which usually occurs early in the disease course—to more advanced stages of hepatic disease, including non-alcoholic steatohepatitis or NASH, cirrhosis, and liver cancer. Simple steatosis can progress to inflammation and necrosis (i.e., to NASH), and subsequently evolve into cirrhosis and hepatocellular carcinoma [13]. The diagnosis of NAFLD is that of exclusion and requires identification of hepatic steatosis by histology (defined as liver fat infiltration comprising >5% of hepatocytes) or imaging (bright liver echo-pattern), and exclusion of secondary causes of CLD, such as alcohol (<30 g/day for men and <20 g/day for women), congenital or autoimmune diseases, viruses, and hepatotoxic drugs [14].

The diagnosis of NAFLD can be achieved by several techniques. Although liver biopsy and demonstration of steatosis and other histology hallmarks remain the gold standard for NAFLD diagnosis, other less-invasive techniques such as ultrasonography (US), computed tomography (CT), and magnetic resonance imaging are widely utilized clinically because of a better safety profile and availability, lesser cost, and good sensitivity and specificity compared to the gold standard [13,15,16]. Moreover, several European associations, including the European Association for the Study of the Liver, recommend US as the first-line diagnostic procedure for NAFLD in most patients [13]. However, accounting for the limitations of each modality is crucial for proper NAFLD diagnosis. For instance, the sensitivity of US for NAFLD decreases when <30% of hepatocytes are involved, but this modality may also lose sensitivity in morbidly obese individuals [15,16].

Several newer noninvasive approaches have been investigated for the diagnosis of NAFLD, and some of them have been introduced in clinical practice (Table 1).

The fatty liver index (FLI) [22] is calculated based on levels of triglycerides, body mass index (BMI), waist circumference, and γ-glutamyl-transpeptidase. A FLI < 30 rules out NAFLD whereas a FLI ≥ 60 rules in hepatic steatosis. Scores between 30 and 59 are considered undetermined. The ultrasound fatty liver index (US-FLI) [29] is a different score based on US parameters: a score > 2 suggests the presence of steatosis. Another accurate method to quantify hepatic steatosis is represented by assessment of the controlled attenuation parameter (CAP) on hepatic Fibroscan [30]. In NAFLD, Fibroscan has also been used to assess liver stiffness, a measure of liver fibrosis, by transient elastography (TE). The NAFLD Fat Score is another score that can be used to predict NAFLD and liver fat content (AUROC = 0.88) [23]. It is based on presence of MetS, T2DM, fasting serum (fs) insulin, fs-aspartate aminotransferase (AST), and AST/alanine aminotransferase ratio.

Staging of fibrosis in NAFLD can also be accurately estimated using the NAFLD fibrosis score (NFS) [27] calculated on the combination of several parameters: age, BMI, altered glucose metabolism, AST/ALT ratio, platelet count, and albumin levels. Significant liver fibrosis (equivalent to F3-F4 fibrosis on liver biopsy) is highly suspected when the NFS is >0.675. This measurement needs a diagnosis of NAFLD done with other tests/assays. Two other methods have been used to assess probability of cirrhosis in NAFLD, including the AST to Platelet ratio index (APRI test) [31,32] and the FIB-4 test [25,33].

## 3. NAFLD as a Systemic Disorder

As numerous compelling evidence shows NAFLD is a systemic disease, rather than confined to the liver, the increasing clinical and economic burden of NAFLD stems from both hepatic and extrahepatic complications. The hepatic complications of NAFLD include NASH, cirrhosis, and hepatocellular carcinoma, and hence it currently represents the second main indication for liver transplantation, projected to become the main one over the next ten years [34,35]. NAFLD affects many other systems and has been shown to increase the risk of developing a number of diseases, including many cardiovascular diseases (CVD), as well as T2DM, chronic kidney disease, and colorectal and other cancers [36,37,38,39,40,41].

CVDs represent the main cause of mortality in patients with NAFLD, accounting for 40–45% of total deaths, followed by extrahepatic cancers (20%) and liver-related complications (10%) [36,42,43,44,45].

## 4. NAFLD and Cardiovascular Disease: Current Understanding

**Figure 1:**
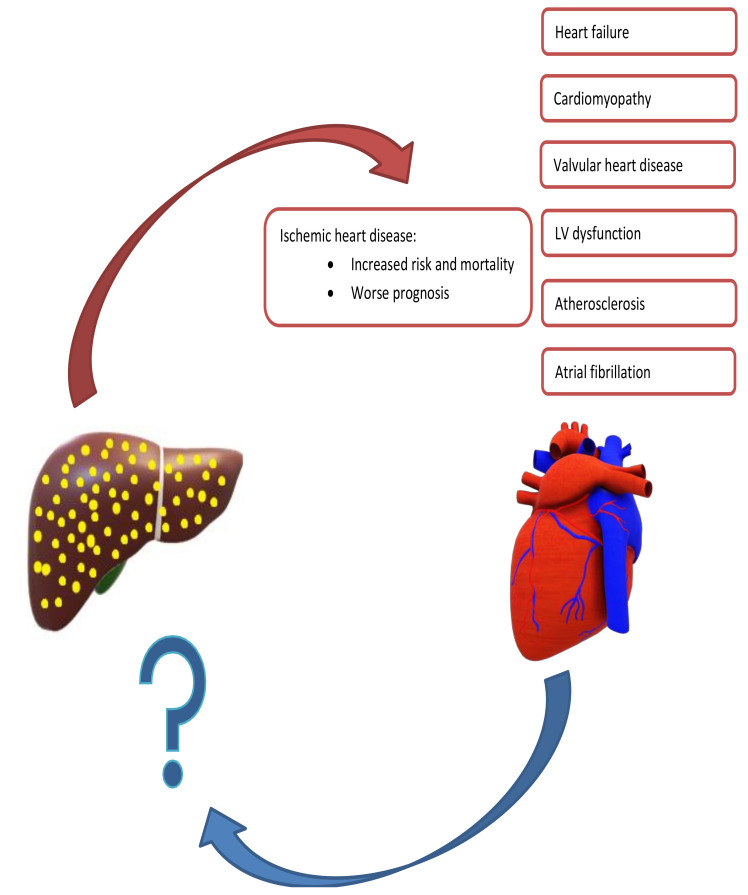
Summary of Mutual Exacerbation of Hepatic and Cardiac Disease Severity. Nonalcoholic fatty liver is associated with several cardiac diseases, including heart failure, cardiomyopathy, ischemic heart disease, and arrythmias. In contrast, there is a paucity of studies evaluating cardiac diseases leading to hepatic dysfunction.

The relationship between NAFLD and CVD is complex (Figure 1). For instance, NAFLD is associated with many CVDs and the total CVD risk is almost double in patients with NAFLD compared to those without [46]. Moreover, as both conditions share many risk factors, most notably T2DM, dyslipidemia, and obesity, it could be conferred that the same factors account for both diseases and the association being merely a result of these common risk factors [47,48]. However, there is ample evidence showing that a direct mechanistic relationship exists between NAFLD and CVD, and that the former is an independent risk factor for the latter [36,41,42,46,48,49,50]. Due to the association with CVD, recent European clinical practice guidelines mandate screening of the CV system in all patients with NAFLD, at least by detailed risk factor assessment and a comprehensive clinical exam [13].

A number of studies suggest NAFLD association with subclinical and clinical CVD, both closely related to atherosclerosis and inflammation. Subclinical CVD is commonly inferred from carotid intima-media thickening (CIMT), increased coronary calcium score (CAC), and abdominal aortic calcification (AAC), whereas clinical CVD includes most commonly ischemic heart disease (IHD) and atrial fibrillation (AF) [41]. In one metanalysis with 3497 subjects, NAFLD was found to be significantly associated with CIMT and NAFLD patients demonstrated a 13% increase in CIMT compared to controls [51]. Similarly, in a large cohort study with 8020 adult men without carotid atherosclerosis (CA) at baseline followed up for eight years, NAFLD regression was associated with a decreased risk of subclinical CA development (HR 0.82) compared to those with persistent NAFLD [52]. The authors explained this association by metabolic factors, which could mediate the effect of NAFLD [52].

Several studies demonstrated an association between NAFLD and CAC, thus implying an increased risk of coronary events in patients with NAFLD [53,54,55,56,57,58,59,60]. In one meta-analysis of 16 cross-sectional studies with 16,433 NAFLD patients and 41,717 controls, it was shown that NAFLD was significantly associated with CAC > 0 and CAC > 100 independent of traditional risk factors [61]. In a second study on 4731 participants with no history of CVD or liver disease and approximately 4 years of follow-up, NAFLD was shown to be associated with CAC development independent of metabolic or CV risk factors [62]. These associations were further established in a recent large meta-analysis on 85,395 participants (including 29,493 patients with NAFLD), where NAFLD was associated with an increased risk of CIMT (OR 1.74), arterial stiffness (OR 1.56), CAC (OR 1.4), and endothelial dysfunction (OR 3.73) [42].

Clinical CVD, most importantly IHD, is the leading cause of death in patients with NAFLD [45,63]. NAFLD patients seem to be at a higher risk of developing IHD [56]. In one study with 445 patients, high risk coronary plaques—assessed by coronary CT angiography—were more likely to occur in patients with NAFLD compared to controls (59.3% vs. 19.0%, respectively) [56]. Similar results were observed in other studies, which concluded higher prevalence of coronary plaques among NAFLD patients independent of other metabolic risk factors [64,65,66]. A striking finding was the observation that higher NAFLD severity directly correlated with higher CVD risk [67]. In one study involving 360 patients with ST-segment elevation myocardial infarction (STEMI), higher NAFLD grade (as assessed by liver US) correlated with higher in-hospital and three-year mortality rates, and the authors concluded recommending NAFLD screening in patients with STEMI [68]. In another study examining 186 non-diabetic patients with STEMI undergone percutaneous coronary intervention (PCI), NAFLD was associated with higher odds of myocardial reperfusion failure, no ST-segment resolution, and higher in hospital major adverse cardiac events (MACE) [69].

AF is the most common sustainable cardiac arrhythmia [36]; the health and economic burden of atrial fibrillation is mainly due to its association with stroke and increased mortality [50]. AF was shown in many studies to be associated with NAFLD [70,71,72,73]. For instance, Targher et al. demonstrated, over a 10-year follow-up, an increased incidence of AF in patients with T2DM and NAFLD compared to patients with T2DM without NAFLD (OR 4.49) [72]. These results were confirmed by a cohort study by Käräjämäki et al. involving 958 hypertensive patients, where they found higher odds of developing AF in patients with NAFLD, independent of T2DM (adjusted OR 1.88) [74].

Several studies suggested NAFLD association with other CVD, including structural and functional cardiac dysfunction and heart valve sclerosis [75]. Some studies reported that NAFLD patients had thicker left ventricular walls during both systole and diastole [76], lower early diastolic relaxation velocity and higher left ventricle (LV) filling pressures [77] and greater LV myocardial mass [78] than patients without NAFLD. In one study assessing cardiac function of 606 T2DM patients according to NAFLD presence, LV diastolic dysfunction was significantly more prevalent in those with NAFLD (59.7% vs. 49%), with stronger association in patients with liver fibrosis independent of insulin resistance and other metabolic risk factors [79]. Moreover, several studies indicated an association between NAFLD and valvular heart disease [80], most commonly aortic valve sclerosis (AVS). In one study with 2212 participants, it was found that patients with NAFLD had 32% higher odds of having AVS than patients without NAFLD, after adjusting for major confounders [81]. This finding was also replicated in T2DM patients with no history of liver or cardiac disease [82,83].

## 5. NAFLD and Cardiovascular Disease: Pathogenic Mechanisms

**Figure 2:**
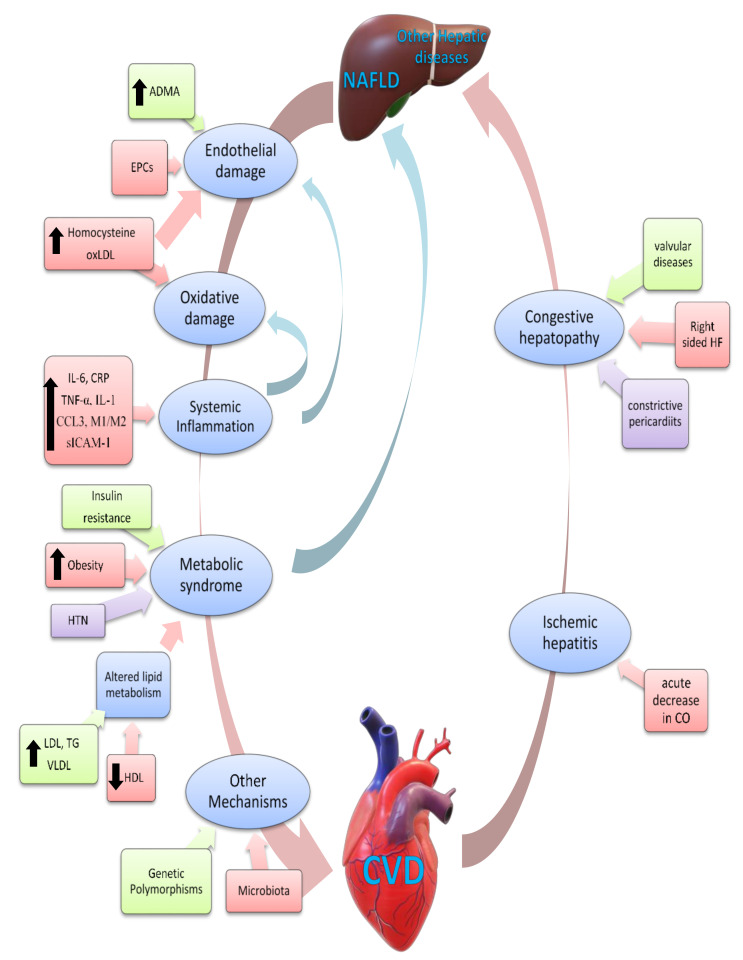
Summary of suggested pathophysiological mechanisms underlying the cardio hepatic interactions in NAFLD, ischemic hepatitis and congestive hepatopathy with cardiovascular diseases. ADMA, asymmetric dimethyl arginine; CCL3, chemokine ligand 3; CO, cardiac output; CRP, C-reactive protein; EPCs, endothelial progenitor cells; HDL, high-density lipoproteins; HF, heart failure; HTN, hypertension; IL-1, interleukin 1; IL-6, interleukin 6; LDL, low-density lipoprotein; M1/M2, macrophage phenotype 1/2 ratio; NAFLD: non-alcoholic fatty liver disease; OxLDL oxidized low-density lipoprotein; sICAM, soluble intracellular adhesion molecule; TG, Triglycerides; TNF-α, tumor necrosis factor α; VLDL, very low-density lipoproteins.

Data on possible mechanisms underlying the interplay between liver and heart in NAFLD are limited and still not completely understood (Figure 2) [84]. Endothelial damage was demonstrated to be an early step towards atherosclerosis, and a number of studies showed that NAFLD is associated with endothelial dysfunction [85,86]. Possible mechanisms for endothelial dysfunction include increased levels of asymmetric dimethyl arginine (ADMA), which is an endogenous antagonist of nitric oxide synthase, which in turn is protective against CVD [87,88]. Moreover, levels of endothelial progenitor cells (EPCs)—which participate in endothelial repair—were shown to be reduced, and attenuated in function, in NAFLD [89].

In addition, NAFLD is associated with increased oxidative stress and altered lipid profile, which in turn may contribute to CVD development [84,90]. It was shown that patients with NAFLD have elevated levels of homocysteine [91,92,93]. Homocysteine, in turn, triggers oxidative stress and endothelial dysfunction, impairs redox status, and enhances platelet activation, all contributing to CV effects [94]. NAFLD severity associates with the extent of serum lipid profile changes, with greater alterations in NASH [84]. Patients with NAFLD have abnormally high triglyceride (TG), very low-density lipoprotein (VLDL), and low-density lipoprotein (LDL) levels, as well as decreased high-density lipoprotein (HDL) levels [95,96,97], a combination resulting in more atherogenic lipid ratios [98]. Similar but more pronounced reduction in HDL and increase in LDL levels were found in NASH, as compared to NAFLD, which hints at similar CV risks [99]. In addition to their levels, lipid composition, subclass, surface apolipoproteins, and phospholipids are important mediators of CV risk [100] and NAFLD severity [97]. Individuals with NASH were shown to have lower levels of larger LDL (LDL1) but increased small dense LDL (LDL3 and LDL4), as compared to those with NAFLD [97].

Insulin resistance, which associates with both CVD and NAFLD, comprises the ability of adipocytes to store fat, resulting in release of free fatty acids into the circulation, and thus exposes the liver to higher levels of free fatty acids [101]. These fatty acids are taken up by the hepatocytes by FATP5 and CD36 receptors, which also get upregulated in obesity [102,103], leading to higher triglyceride synthesis and impaired insulin signalling [104]. Hepatocytes contribute themselves to steatosis accumulation by de novo lipogenesis (DNL), the enzymes of which are upregulated by insulin and glucose [105]. DNL can contribute as much as 25% of the hepatic lipid stores and is believed to be an important contributor of NAFLD development [106,107]. Specifically, 40% of the lipid that builds up in steatosis derive from dietary sugar and fat with the remaining 60% deriving from dysfunctional adipose tissue [106]. 

The liver is an essential player in systemic inflammation and immunity. For instance, it harbors the largest number of resident macrophages along with a high number of several other immune cells [108]. It also generates and interacts with various inflammatory hormones/cytokines secreted from places like visceral adipose tissue, macrophages, and endothelial cells, which are associated with CVD initiation and progression [109,110]. Higher pro-inflammatory states associate with worse metabolic, histological, and hemodynamic features in NAFLD [111]. C-reactive protein (CRP), an inflammatory marker mostly produced in the liver, has been implicated as an independent risk factor for CVD in many studies [112]. Compared to patients with no steatosis, patients with NASH were found to have significantly higher levels of CRP, fibrinogen, and plasminogen activator inhibitor-1 (PAI-1) activity. NASH severity on histology correlated independently of other variables with levels of these markers [113].

Diseased liver was shown to secrete increased levels of cytokines systemically, which are associated with CVD and drive systemic inflammation [84,114]. Indeed, patients with NAFLD were shown to have increased markers of systemic inflammation including interleukin 6, high sensitivity CRP, interleukin 1b, tumor necrosis factor [TNF]-α, chemokine [C-C motif] ligand 3, soluble intracellular adhesion molecule 1, and macrophage phenotype 1/2 ratio [M1/M2] [111,115,116,117,118]. Systemic inflammation increases CVD risk by causing endothelial dysfunction and oxidative stress, and altering vascular tone [114,119]. In one study, NASH patients were found to have increased expression of several genes linked to inflammation and plaque formation, as compared to simple steatosis [120].

Overall, these studies show that the observed systemic inflammation and immune dysfunction are directly contributed to by the liver, and this chronic inflammation drives CVD initiation and/or progression.

Similarly, the liver is a very important, if not exclusive, source of both pro- and anticoagulant factors [121]. Several studies demonstrated increased pro-coagulant and decreased anticoagulant factor production in NAFLD [122,123], which is one mechanism postulated to link CVD and NAFLD. Kotronen et al. demonstrated that the activity of factors VIII, IX, XI, and XII were increased in patients with NAFLD, independent of other variables like BMI and age, as compared to those without NAFLD [122].

Furthermore, several studies investigated plasminogen inhibitor activator 1 (PAI-1) level, which is secreted by the liver and functions to inhibit fibrinolysis and thus activate the coagulation system, leading to an increased atherosclerotic state [123]. Liver steatosis was shown to be an independent determinant of PAI-1 levels, with levels of this factor progressively rising with increasing degrees of steatosis [124]. Another study by Verrijken et al. showed that fibrinogen, factor VII, von Willebrand factor and PAI-1 were increased, while antithrombin III was decreased. Interestingly, PAI-1 levels were significantly correlated with histological severity of NAFLD [125]. In another study by Song et al., the authors suggested a direct association between PAI-1 levels and coronary heart disease risk [126]. Increased pro-coagulant and decreased anti-coagulant factors boost risk for atherosclerosis and CVD [127].

Recent evidences point to a significant role of the intestinal microbiota in NAFLD pathogenesis and progression. In their study, Loomba et al. analyzed the gut microbiota of 86 patients with biopsy-proven NAFLD and demonstrated a positive association of specific bacterial species, such as *Proteobacteria phylum*, with progression of NAFLD from mild/moderate to advanced fibrosis. They also used data to construct a robust model that was able to accurately (AUROC 0.936) diagnose advanced fibrosis [128]. Other studies similarly demonstrated increased *Proteobacteria phylum* with increased Enterobacteriaceae and decreased Rikenellaceae and Ruminococcaceae families in patients with NAFLD or NASH compared with healthy controls [129]. Moreover, the gut microbiome can systemically secrete several molecules such as secondary bile-acids, trimethylamine (TMA), and short chain fatty acids [84]. These molecules were demonstrated to affect energy balance and insulin sensitivity and thus could affect both NAFLD and CVD [130]. A clearer link is found between CVD, NAFLD, and trimethylamine-N-oxide, which is thought to be a pro-atherogenic compound [131,132]. Furthermore, incretins such as glucagon like peptide 1 (GLP-1) are hormones secreted by the gastrointestinal tract and function in regulating postprandial glucose metabolism [84]. The cardioprotective effects of GLP-1 are well described [133] and GLP-1 agonists such as exenatide were shown to improve NASH, vessel inflammation, and plaque size [134]. Despite these results, large discrepancies are found with divergent results for phylum, family, genus, and species across many studies investigating microbiome and NAFLD [129].

Genetic polymorphisms were also studied as predisposing factors for NAFLD, and several alleles were found to be associated with NAFLD and the progression of hepatic fibrosis. In one meta-analysis by Singal et al., a single nucleotide polymorphism—PNPLA3—was associated with increased risk of fibrosis (OR 1.23) and HCC (OR 1.67) in patients with NAFLD [135]. In another study, Liu et al. demonstrated an association between NAFLD and the stage of hepatic fibrosis with TM6SF2 allele, independent of PNPLA3 [136]. Other genetic variants including MBOAT7 and glucokinase regulatory protein were suggested to be associated with increased risk of steatosis, NASH and increased fibrosis stage [137,138]. Much less is known regarding the association of these alleles with CVD. In one twin study investigating heritability of NAFLD and associated CVD, a correlation was established between hepatic steatosis and monozygotic, rather than dizygotic twins, even after adjusting for other possible confounders [139]. However, another twin study failed to demonstrate heritability of NAFLD or its associated CVD [140]. Moreover, in several studies, mutation in TM6SF2 was associated with LDL, VLDL, and TG reduction [141,142,143,144,145]. Indeed, in a recent large exome-wide association study including >300,000 individuals, both polymorphisms in TM6SF2 and PNLPA3 were associated with lower lipid levels and a lower risk of CAD, but an increased risk of fatty liver and T2DM [146]. A finding also confirmed with a 4081 adult cohort followed up for 11.3 years, where PNPLA3 allele was found to be associated with a fourfold increase in the hazard of liver disease-related mortality but reduced risk of death from CVD and overall mortality [147].

## 6. Nonclinical Experimental Studies on CVD and NAFLD Association

The associations between heart disease and metabolic steatosis were also demonstrated in animal models. In rodents, a high-fat diet resulted in hepatic steatosis and up-regulation of NF-kB, which increased hepatic production of inflammatory cytokines such as IL-6, IL-1β, and TNF-α, along with activation of Kupffer cells and macrophages [148]. The role of TNF-α in NAFLD initiation and progression was also investigated in mice. For that purpose, Kakino et al. analyzed NAFLD progression in TNF-α knockout NAFLD mice models, where they found that these mice significantly regressed steatosis and fibrosis and improved glucose tolerance at 20 weeks, as compared to wild NAFLD mice [149]. Moreover, the effect of IL-1β on steatosis was also studied, where in one study, IL-1 depletion from liver Kupffer cells in obese mice significantly reduced hepatic steatosis, triglyceride levels, and lipogenic enzyme expressions with similar results achieved with utilizing IL-1 receptor antagonists [150].

Moreover, and similar to data from clinical studies, plasma PAI-1 levels in mice with NAFLD were shown to be significantly elevated and associated with the degree of steatosis and hepatic expression of PAI-1 [151]. It was also shown that PAI-1 modulates the development of atherosclerosis in mice [152,153] and that obese mice had around 2-fold higher PAI-1 liver expression than lean mice [154]. Other mechanisms of cardiac disease in NAFLD spectrum includes changes in collagen isoforms expression in the heart. One study demonstrated that cirrhotic rats had increased type 1 collagen content, leading to increased ventricular stiffness and diastolic dysfunction, as compared to sham animals [155].

The effect of some medications was also investigated in animals with steatosis. In one such study, angiotensin-II receptor blocker (ARB) Losartan’s effect was studied in mice models of NASH. Results showed that after eight weeks of treatment, ARB inhibited liver fibrosis development along with suppression of activated hepatic stellate cells and TGF-β and also suppression of TLR4 and NF-κB expressions [156]. In another study, the effect of nonsteroidal anti-inflammatory drugs (NSAIDs) was investigated in mice NASH models. After six months of treatment, mice who received Aspirin had significantly reduced steatosis, and all four tested NSAIDs (meclofenamate, mefenamate, flufenamate, and aspirin) were able to stop steatosis progress to steatohepatitis [157].

## 7. Effect of Cardiovascular Diseases and Their Treatments on NAFLD

Emerging data on the interplay between NAFLD and CVD show a complex two-way relationship between the two conditions [158,159,160,161]. On one hand, and as previously stated, NAFLD was suggested to be a major risk factor for many CVD [44,45,52,66]. On the other hand, other studies investigated the reverse relationship: the effect of cardiac pathology, mainly acute and chronic heart failure, on hepatic disease [158,159,160,161]. Liver disease caused by heart failure includes congestive hepatopathy, or chronic hepatic passive congestion, and cardiogenic ischemic hepatitis, a rapid and acute cardiogenic liver injury [159].

Due to the strong association between NAFLD and CVD, and the increased risk of mortality from CV events of patients with NAFLD, a number of primary and secondary prevention strategies were recommended by the American College of Cardiology, the American Heart Association, the European Association for the Study of the Liver, and the Italian Association for the Study of the Liver. Primary prevention strategies for NAFLD also target the traditional risk factors for CVD: healthy dietary pattern, moderate or vigorous exercise, and optimal body weight achievement [13,162,163].

Lifestyle modifications remain a cornerstone in both the treatment of NAFLD and the prevention of CV complications, considering that there is a lack of definitive treatments [164]. For instance, in one prospective study on 293 patients with NASH, lifestyle modifications that are beneficial for CVD (decrease in calorie intake and increase in exercise) achieved regression of fibrosis and resolution of steatohepatitis in 19% and 25% of patients, respectively [165]. To date, a number of drugs have been studied as possible treatments of NAFLD and secondary prevention of CV complications [164]. For example—whose CV benefits are clearly established—were shown to be inversely correlated with NAFLD and liver fibrosis (OR 0.57 and 0.47, respectively) [166]. In another study with 1600 patients over three years, atorvastatin was shown to improve liver function tests and decrease cardiovascular events (from 10 to 3.2 events per 100 patient-years) in NAFLD patients who took it compared to those who did not [167].

Other drugs including metformin, thiazolidinediones (TZD), and aspirin have been investigated as potential NAFLD treatments. In one meta-analysis of randomized controlled trials (RCTs) involving a total of 417 participants, metformin was shown to improve liver function in patients with NAFLD, but failed to significantly improve histological response [168]. Moreover, metformin was shown to be significantly associated with weight loss, improved insulin sensitivity [169] and lipid profiles (decreased LDL and increased HDL levels) in NAFLD patients [170,171], which might in turn decrease the CV risk related to NAFLD.

In another meta-analysis including 8 RCTs and a total of 516 patients evaluating TZD effect on histology of biopsy-proven NASH, TZD treatment (5 RCTs evaluating pioglitazone; 3 evaluating rosiglitazone) was associated with improved fibrosis (OR 3.15) and NASH resolution (OR 3.22)—a trend seen in patients with or without T2DM [172]. However, TZD therapy was associated with weight gain and limb edema [169,172], and because of the short duration of the trials, there was no report of congestive heart failure or increased CV mortality [169]. RCTs on TZD therapy, in general, had small sample sizes, short duration, and evaluated the impact of TZD on histological rather clinical response [172,173].

In a recent prospective cohort study with 361 adults with biopsy-confirmed NAFLD, with nine-year follow-up, daily aspirin use was associated with significantly lower odds for NASH (OR 0.68) and fibrosis (OR 0.54), with greatest benefit with at least four years of aspirin use—an association not seen with other non-aspirin NSAIDs [174]. Furthermore, a cross-sectional study including 11,416 patients showed an inverse correlation between regular aspirin use (defined as ≥15 times in the prior month) and prevalent NAFLD (OR 0.62), although this was limited to older men (>60 years) [175]. Aspirin, and not ibuprofen use, showed association with lower indices of liver fibrosis among adults with chronic viral hepatitis, suspected alcoholic liver disease, and NAFLD [176]. The suggested mechanisms of action of platelets in NAFLD stem from the growing evidence of the active role of platelets in liver disease and inflammation [177,178,179]. Indeed, in a model of viral hepatitis, activated platelets were found to contribute to cytotoxic T-lymphocyte-mediated liver damage [180,181]. Moreover, blocking platelet activity with drugs such as aspirin or clopidogrel hampered T cell influx and subsequent liver damage and tumorigenesis in viral hepatitis [182]. Another recently suggested role for platelet inhibition by aspirin in NAFLD is activation of the PPAR*δ*-AMPK-PGC-1*α* pathway, which in turn inhibits lipid synthesis and elevates catabolic metabolism, and modulation of mannose receptor and CCR2 in macrophages, all of which are suggested to ameliorate NAFLD and atherosclerosis [183]. Despite the favorable results these studies show, further investigations with larger sample sizes are required to assess the impact of these drugs, among others, on NAFLD inception and progression.

Another interesting topic to be addressed is the effect of newer anti-diabetes and anti-hypertensive drugs on NAFLD inception or progression, with or without concomitant CVD. GLP-1 receptor agonists (GLP-1Ras) and Renin-Angiotensin-Aldosterone System Inhibitors (angiotensin converting enzyme inhibitors (ACEi) and angiotensin receptor blockers (ARBs)) were investigated as potential treatments for NAFLD. Early studies demonstrated decreasing and normalizing AST levels in T2DM patients receiving exenatide with elevated AST levels at baseline, compared to those who did not receive it [184]. Recent meta-analyses showed association of GLP-1Ras, mainly exenatide and liraglutide, with reduced body mass index (BMI) and waist circumference (WC) [185,186], and liver fat fraction [185]. Moreover, in subgroup analyses, patients receiving exenatide had improvements in liver enzyme levels (AST and ALT) [185]. In a recent clinical trial, the LEAN (Liraglutide Efficacy and Action in NASH) by Armstrong et al., 52 overweight patients with NASH were randomized to receive liraglutide or placebo (26 in each group). The results of this small pilot study showed that patients in the experimental treatment arm had 4.3 times higher chance of histologically-proven NASH resolution [187].

SGLT2 inhibitors are another class of diabetes and CVD drugs with recent implications in NAFLD treatment. In one phase III randomized controlled trial by Frías et al., 695 patients with T2DM were randomized to receive exenatide plus placebo, dapagliflozin plus placebo or a combination of the two drugs and followed up for 28 weeks. The results of this study showed that all groups had a decrease in the traditional CV risk factors, such as blood pressure, HbA1c and glucose levels, that were more pronounced and superior in the group receiving both drugs [188]. A recent post hoc analysis of the same study evaluated the effects of the tested therapies on non-invasive markers of hepatic steatosis (FLI, NAFLD Fat Score, FIB-4 index, NAFLD Fibrosis Score and liver enzymes) and found that both drugs had stronger effects than each drug alone in ameliorating markers of hepatic steatosis and fibrosis in patients with T2DM [189]. Another study showed that NAFLD patients with T2DM who received dapagliflozin alone had a significant decrease in CAP, liver stiffness, and AST and GGT levels compared with controls [190]. Other studies also demonstrated the potential of dapagliflozin monotherapy to reduce liver fat assessed by MRI, liver injury biomarkers such as enzyme levels [191], and achieve histological improvement with fibrosis regression [192] in NAFLD patients with T2DM.

RAAS activation was shown to be upregulated in NAFLD [193] and to play a role in development of inflammation and insulin resistance [194], both of which are possible risk factors for NAFLD. Pellusi et al. studied the effect of ACEi and ARBs in an observational cohort of 118 diabetic patients with a median follow-up of 36 months. The authors found that treatment with ACEi or ARBs was associated with decreased histological fibrosis progression [195]. Other studies employing ARBs found improvement in both AST levels and histological stage of patients with NAFLD [196,197]. Moreover, authors investigated the role of selective mineralocorticoid receptor (MR) antagonists, such as spironolactone and eplerenone. Indeed, some clinical trials found that combined low-dose spironolactone plus vitamin E decreased NAFLD liver fat score, an index of steatosis, along with insulin levels and homeostasis model assessment of insulin resistance (HOMA-IR) [198,199]. In addition, eplerenone was suggested to prevent NASH development and improve metabolic abnormalities in mice by inhibiting inflammatory responses in both Kupffer cells and macrophages [199,200].

Beta blockers (BB)—a heterogeneous class of drugs—are commonly used for multiple CVD and other medical indications. BB are known to worsen metabolic parameters: increase weight [201], fasting glucose levels, TG, LDL, and decrease HDL and energy expenditure [202,203,204,205,206,207,208,209] with most of these adverse events observed with the conventional non-selective BB with no intrinsic sympathomimetic activity [210,211]. However, these adverse metabolic side-effects were not shown in newer generation BB with vasodilator properties such as carvedilol and nebivolol [212,213]. The role of BB in NAFLD remains unclear and inconclusive due to scarcity of data. In one study on mice as NASH models, propranolol seemed to enhance liver injury as evident by higher necrosis scores in mice that took it and activate the cell-death pathway by increasing the release of lactate dehydrogenase, FAS-L, and TNF-α [214]. In another study on rats as NAFLD models, carvedilol was shown to improve liver enzymes, lipids, and histology [215].

Bariatric surgery—an effective treatment strategy for severe obesity—has been shown to cause a significant decrease in liver transaminases and histology improvement in NAFLD patients [216]. Moreover, in one prospective study on 109 morbidly obese patients with NASH who underwent bariatric surgery, NASH disappeared in 85% of them and levels of liver transaminases significantly decreased [217]. Moreover, bariatric surgery was shown to improve the traditional CV risk factors. In a systematic review of 73 studies including a total of 19,534 patients with about 58 months of follow-up, 73% of subjects had improvement in T2DM, 65% in hyperlipidemia, and 63% in hypertension [218].

Vitamin E was also studied as a potential treatment for NAFLD due to its anti-oxidative properties. In one meta-analysis of three trials analyzing vitamin E supplementation in 242 patients with NASH, vitamin E use was associated with improved ALT levels, steatosis, lobular inflammation, and ballooning but not fibrosis of the liver [219]. However, vitamin E supplementation was shown in other studies to have no apparent effects on CV outcomes [220].

Other drugs, including obeticholic acid, saroglitazar, and elafibranor are currently being investigated for NAFLD in large clinical trials. Obeticholic acid is a bile acid derivative that can bind to and activate farnesoid X receptors, which in turn can increase insulin sensitivity, decrease hepatic gluconeogenesis, and protect against cholestasis liver injury [221,222]. In one trial on 283 patients with NASH, those taking obeticholic acid were more likely to have improved liver histology than those taking placebo at 72 weeks (RR 1.9) [223].

Elafibranor is a dual PPAR-α/δ agonist—both receptors being implicated in the activation of inflammatory changes within the liver [224,225]. In a preliminary study, Ratziu et al. observed that elafibranor resolved NASH without fibrosis worsening [226]. Saroglitazar is a dual PPARα/γ agonist indicated mainly for the treatment of diabetic dyslipidemia and hypertriglyceridemia not controlled by statins [227]. This drug is currently being investigated as a potential treatment for NAFLD in an ongoing phase 2 trial [224]. Further studies are needed before more stringent recommendations can be done on the use of obeticholic acid, saroglitazar, and elafibranor.

The relationship between CVD treatments and NAFLD can also encompass a potentially detrimental effect of some molecules on liver disease inception or progression. Indeed, several CV drugs have been linked to hepatic steatosis, both microvesicular and macrovesicular. Drugs potentially causing microvesicular steatosis include aspirin, nonsteroidal anti-inflammatory drugs (NSAIDS), and valproic acid, among others, which can lead to lipid accumulation in hepatocytes and subsequent steatosis [228]. On the other hand, drugs implicated in macrovesicular steatosis include amiodarone, a frequently used antiarrhythmic agent with a wide range of hepatic and thyroid adverse events. Up to 30% of patients taking amiodarone can develop elevated liver enzymes, with a proportion of 1–2% developing overt steatohepatitis [228]. Furthermore, a recent meta-analysis concluded that patients taking amiodarone have a higher risk of hepatic adverse events (RR 2.27), even at low doses, as compared to placebo [229].

## 8. Effect of NAFLD on Progression of CVD

In addition to the association of NAFLD with CVD onset, a role has been hypothesized for NAFLD on the progression of CVD, in terms of development of cardiomyopathy (both ischemic and tachycardia-related cardiomyopathy), heart failure (HF) and worsening HF stage.

Despite the growing evidence showing association of NAFLD with CVD, the relationship is still uncertain for many reasons [48,53]. First, a number of studies, two of which carried out by Kim et al. and Lazo et al., featuring a large number of participants (*n* = 11,154 and 11,371, respectively), concluded that there was no association between increased CV mortality and NAFLD [230,231]. Second, conclusions of meta-analyses have been questioned because of the variability of the included articles in these studies, mostly related to the different diagnostic modalities utilized for NAFLD diagnosis [232]. Moreover, the observational nature of the individual studies demonstrating association between CVD and NAFLD leaves room for confounding bias; and thus interpreting these results require caution and does not allow to draw definitive causal inferences [43,232].

Lastly, and to our knowledge, no study questioned the risk of developing NAFLD in patients presenting with a first episode of IHD or AF. Meanwhile, articles focusing on liver disease caused by cardiac pathology included only congestive hepatopathy and ischemic hepatitis, both well described in the literature. Moreover, we could not find a study quantifying the de novo incidence of NAFLD in patients with IHD as most articles investigate the reverse relationship: incidence of IHD/CVD in patients with known NAFLD.

## 9. Conclusions

The association of NAFLD with the CV system is quite complex and incompletely understood. NAFLD is suggested to be implicated in many CVDs and seems to worsen the prognosis of patients with CVD. On the other hand, the effect of CVD on liver pathology is not well studied. No studies to our knowledge investigated a possible relationship between NAFLD inception and progression due to CVD or the incidence of NAFLD in patients with CVD. Moreover, as there is no definitive treatment for NAFLD yet, many CV drugs show promising results in NAFLD treatment both biochemically and histologically with fibrosis regression; others were shown to be associated with increased steatosis. Despite the suggested results, further studies are needed to better understand the two-way liver-heart interplay and the roles of drugs in the pathophysiology and treatment of NAFLD.

## Figures and Tables

**Table 1:** Current strategies to diagnose and stage NAFLD.

Test	Type	Invasiveness	Accuracy ^ in Steatosis Detection	Liver Fat Detection	Liver Fibrosis Staging	Reference
Liver biopsy	Histology	+++	>99%	✓	✓	[17]
MRI-PDFF	Imaging	-	98%	✓	✓	[18]
US	Imaging	-	91% *, 93% **	✓	✓	[19]
DGE-MRI	Imaging	+	83% *, 94% ***	✓	✓	[20]
CT	Imaging	+	67% *, 90% ***	✓	✓	[20]
US-FLI	Imaging	-	90%	✓	✕	[21]
Fatty Liver Index	Score based on biochemical parameters	-	84%	✓	✕	[22]
NAFLD Fat Score	Score based on biochemical parameter	-	76%	✓	✕	[23]
CAP	Imaging	-	76%	✓	✕	[24]
Fib-4	Score based on biochemical parameters	-	90%	✕	✓	[25]
VCTE	Imaging	-	89%	✕	✓	[26]
NAFLD Fibrosis Score	Score based on biochemical parameters	-	86%	✕	✓	[27]
APRI	Score based on biochemical parameters	-	48%, 66%	✕	✓	[28]

APRI, AST to Platelet ratio index; CT, Computed tomography; DGE-MRI, dual gradient echo magnetic resonance imaging; US, Ultrasonography; MRI-PDFF, MRI-based proton-density fat fraction; US-FLI, ultrasonographic fatty liver indicator; VCTE, Vibration-controlled transient elastography; CAP, Fibroscan Controlled Attenuation Parameter. **^** Accuracy was calculated as Accuracy = ((Sensitivity) * (Prevalence)) + ((Specificity) * (1 − Prevalence)) based on data derived from the referenced publications. * detection of ≥5% of steatosis ** detection of ≥10% of steatosis *** detection of ≥30% of steatosis.
